# Working Memory, Language Skills, and Autism Symptomatology

**DOI:** 10.3390/bs2040207

**Published:** 2012-11-02

**Authors:** Jillian M. Schuh, Inge-Marie Eigsti

**Affiliations:** 1 Division of Neuropsychology, Department of Neurology-FWC, Medical College of Wisconsin, 9200 West Wisconsin Ave, Milwaukee, WI 53226, USA; 2 Department of Psychology, University of Connecticut, 406 Babbidge Road, U-1020, Storrs, CT 06269, USA; E-Mail: inge-marie.eigsti@uconn.edu

**Keywords:** Autism, working memory, executive functioning, language

## Abstract

While many studies have reported working memory (WM) impairments in autism spectrum disorders, others do not. Sample characteristics, WM domain, and task complexity likely contribute to these discrepancies. Although deficits in visuospatial WM have been more consistently documented, there is much controversy regarding verbal WM in autism. The goal of the current study was to explore visuospatial and verbal WM in a well-controlled sample of children with high-functioning autism (HFA) and typical development. Individuals ages 9–17 with HFA (*n *= 18) and typical development (*n *= 18), were carefully matched on gender, age, IQ, and language, and were administered a series of standardized visuospatial and verbal WM tasks. The HFA group displayed significant impairment across WM domains. No differences in performance were noted across WM tasks for either the HFA or typically developing groups. Over and above nonverbal cognition, WM abilities accounted for significant variance in language skills and symptom severity. The current study suggests broad WM limitations in HFA. We further suggest that deficits in verbal WM are observed in more complex tasks, as well as in simpler tasks, such as phonological WM. Increased task complexity and linguistic demands may influence WM abilities.

## 1. Introduction

The autism spectrum disorders (ASD) are a set of severe neurodevelopmental disorders characterized by striking impairments in social interaction, language and communication, and the presence of restricted and repetitive behaviors and circumscribed interests. Some researchers have proposed that deficits in executive functions, which allow for the allocation of attentional and cognitive resources, may underlie and influence this triad of impairments [1,2]. Supporting this, a wide range of executive functioning deficits has been noted in ASD (for reviews, see [3,4]). An essential feature of executive functioning is the ability to maintain and update representations over time, a process known as working memory (WM). While some previous research on ASD links social skills to WM [5], there is still controversy about the presence or specificity of WM impairments in ASD. Many studies find deficits [6,7,8,9,10] while others do not [11,12,13]. 

Several factors may contribute to this discrepancy. First, impairments may differ across WM modalities; deficits have been reported for phonological processing [14,15] and spatial WM (e.g., [16,17]), but not verbal WM (e.g., [16,17]). Second, complexity of WM demands may influence performance. WM serves as a limited capacity system that involves storage aspects as well as the processing, or manipulation, of information [18]. Research on WM in typical development suggests simple tasks may rely more heavily on the holding, or temporary storage, responsibilities of WM, whereas complex tasks may draw upon both storage and maintenance aspects of WM [19,20]. WM in ASD may be intact for simple tasks that require holding of information only (e.g., digit span), although impairments often emerge on more complex tasks that involve executive control (e.g., recall or spatial reversal; see [21] for review).

Performance is also impacted by sample characteristics, such as age, cognitive and language ability, and symptom severity. Preschool children with ASD have been found to perform similarly to age-matched controls on tasks of spatial WM [13], suggesting WM impairments may be less salient early in development. Additionally, intellectual abilities appear to influence WM performance (e.g., [22]). While the majority of WM studies in ASD to date focus on high-functioning autism (for review, see [21]), some include Asperger’s disorder whereas others do not. This diagnosis is characterized by less language impairment [23] and may result in a different profile for WM skills. Low-functioning ASD is also typically characterized by WM deficits, but not an exacerbation relative to IQ [21]. Finally, because WM skills are closely intertwined with language processing [24], including individuals with heterogeneous language abilities may obscure WM deficits in ASD.

This study addressed the need to assess WM abilities in a well-characterized and homogeneous sample of children and adolescents with high-functioning autism, compared to a sample of typically-developing peers. By carefully matching for factors including chronological age, nonverbal IQ, and language level, the present study provides insight into the nature of WM impairment in ASD, using WM tasks of varying complexity and modality. Because spatial WM deficits have been consistently noted in ASD, the current study primarily focused on both simple and complex verbal WM measures, although it also included a spatial WM task. It was hypothesized that children and adolescents with ASD would show impairments in spatial WM and also aspects of verbal WM, particularly for those with the most complex task demands.

## 2. Results and Discussion

### 2.1. Group Comparisons

**Figure 1:**
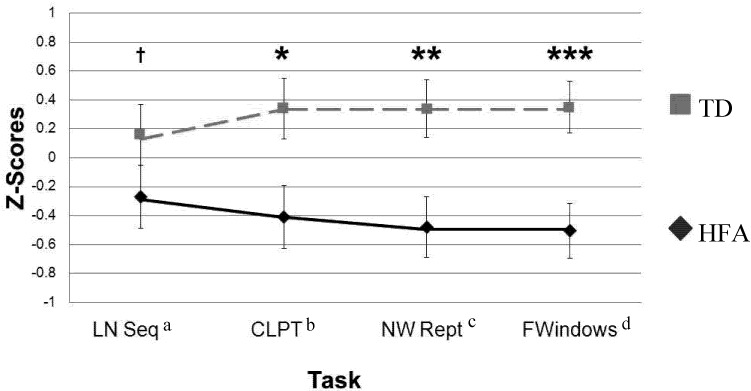
Working memory abilities as a function of diagnostic group. Graph excludes two outliers for Letter-Number Sequencing. ^†^* p *< 0.10; * p < 0.05; ** p < 0.01; *** p < 0.001. ^a^ Letter-Number Sequencing; ^b^ Competing Language Processing Task; ^c^ Non-word Repetition; ^d^ Finger Windows.

To permit comparison across domains, measures of WM were transformed to z-scores that were calculated using the total sample; both standard scores and z-scores are shown in Table 1. Data were examined for outliers; data from two HFA participants from the Letter-Number Sequencing subtest were excluded as they performed more than two standard deviations from the HFA group mean (1 lower, 1 higher). A 2 (group) × 4 (WM task z-scores) repeated measures ANOVA indicated a significant main effect of group, *F *(2, 36) = 26.69, *p *< 0.001, *η*^2^*_p_* = 0.46, with the TD group scoring higher than the HFA group across all WM measures. There was no main effect of task, *F *(2, 36) = 0.05, *p* = 0.98, *η*^2^*_p_* = 0.002, and no significant task by group interaction, *F *(2, 36) = 1.19, *p* = 0.32, *η*^2^*_p_* = 0.04; findings are shown in Figure 1 [25].

**Table 1:** Performance on working memory measures by group.

	HFA; *n* = 18		TD Controls; *n *= 18		*F*	*p*	*h^2^_p_*
	Score	Z-Score	Score	Z-Score			
*Spatial WM:*							
Fing Wind SS ***	9 (2); 5–14	−0.50 (0.93); −2.08–1.47	11 (2); 6–14	0.35 (0.73); −1.69–1.47	14.18	<0.001	0.30
*Complex Verbal WM:*							
Let-Numb Seq SS ^†^ (*n* = 16 for HFA)	10 (3); 5–17	−0.27 (0.85); −1.76 – 1.87	11 (2); 9–16	0.16 (0.53); −0.55–1.57	3.09	0.09	0.09
Comp. Lang. Proc. RS *	31 (16); 10–63	−0.41 (0.98); −1.90- 1.41	44 (14); 16–63	0.34 (0.90); −1.62–1.82	4.88	0.03	0.13
*Phonological WM:*							
Nonword Rep. RS **	16 (2); 12–20	−0.48 (1.08); −2.54–1.35	18 (1); 15–20	0.34 (0.54); −0.60–1.02	7.98	0.01	0.20
*WM Composite *^a^^,^***		−0.44 (0.71); −1.38–1.48		0.29 (0.38); −0.33–0.93	15.44	<0.001	0.32

Notes: ^†^* p* < 0.10; * p < 0.05; ** p < 0.01; *** p < 0.001. Data presented as *Mean* (*SD*); Range. Results are raw scores (RS) or standard scores (SS), with a *M* = 10, *SD* = 3, as indicated. Fing Wind = Finger Windows; Let-Numb Seq. = Letter-Number Sequencing; Comp. Lang. Proc. = Competing Language Processing (maximum score = 72); Nonword Rep = Non-Word Repetition (maximum score = 20). ^a^ Composite calculated from z-scores in Letter-Number Sequencing, Competing Language Processing Test, and Non-Word Repetition.

In addition to the previous examination of group differences, the data were also probed to reveal clinical levels of competence on WM tasks. That is, for those WM tasks that include standardized normative data, Finger Windows and Letter-Number Sequencing, performance within each group was examined to indicate the relative proportion of individuals scoring below, at, or above average, relative to national normative samples. Within the HFA group, there were 13 scores in the low average or impaired range, contributed by 11 different children (out of 18; 61%), compared to a single such score in the TD group, as shown in Table 2. One participant with HFA had scores in the “impaired” range for two WM tasks. 

**Table 2:** Number of individuals in each group per clinical range for working memory tasks.

	Impaired	Low Average	Average	High Average	Superior
	TD: HFA	TD: HFA	TD: HFA	TD: HFA	TD: HFA
*Spatial WM: *Finger Windows	1:2	0:7	9:6	7:2	1:1
*Complex Verbal WM:* Letter-Number Sequencing	0:2	0:2	12:10	5:1	1:3
*Total*	1:4	0:9	21:16	12:3	2:4

Notes: TD = Typical Development; HFA = High Functioning Autism. Range for standard scores (SS) is as follows: Impaired (<6), Low Average (7–8), Average (9–11), High Average (11–13), Superior (>14).

Overall, individuals with HFA demonstrated marked WM impairment in both verbal and non-verbal domains. This is particularly striking given that all participants in the HFA group had IQ scores in the average or above average range, and suggests WM limitations may be disproportionate to overall cognitive functioning. This also suggests that, in addition to group differences, the difficulties in WM task performance in this study likely reflect real-life functional impairment in a significant subset of individuals. This may help to explain, for example, why some individuals with HFA struggle with more complex academic work, despite general learning and memory abilities being intact.

### 2.2. Regression Analyses

Because there were no differences in performance across the WM tasks, a WM composite was created, which included z-scores averaged across all WM tasks. The relative impact of overall WM ability on language and symptom severity for the HFA group only was explored using hierarchical multiple regression. In all regression analyses, predictors were entered in two steps. Stanford-Binet-5 nonverbal IQ was entered in the first step in all analyses, to control for general cognitive ability, and the WM composite was added in the second step. Statistics are presented in Table 3. A first analysis examined language (CELF-4 Core) as a dependent variable. There was a trend for overall WM ability, but not nonverbal cognition, to account for a significant proportion of the variance in language abilities. Two additional regression analyses explored the impact of WM capacity on symptom severity. In the first regression, testing SRS scores as a predictor, both nonverbal IQ and WM capacity predicted symptom severity. In the second regression, testing ADOS Social + Communication Domains (summed score) as a predictor, WM ability but not nonverbal IQ accounted for significant variance in symptom severity in the HFA group. A third regression, not reported here, examined IQ as a predictor of WM composite scores across the two groups. Results indicated that IQ just missed significance as a predictor of WM ability, *F *(1, 36) = 4.00, *p *= 0.06. Taken together, WM capacity, above and beyond nonverbal IQ, accounted for a significant portion of the variance in language ability and symptom severity.

**Table 3:** Hierarchical multiple regression models of working memory ability predicting language and autism symptom severity.

		Language a		SRS b		ADOS b	
		Step 1 (NVIQ) β	Step 2 (WM) β	Step 1 (NVIQ) β	Step 2 (WM) β	Step 1 (NVIQ) β	Step 2 (WM) β
HFA Group Only; *n *= 18							
	Constant	100.990 ***	101.221 ***	100.596 ***	93.752 ***	4.520	4.973
	Stanford Binet NVIQ	0.070	0.118	−0.564	−0.522 *	0.504	0.213
	WM Composite	-	0.601 *	-	−0.634 *	-	−0.616 **
*R^2^*		0.005	0.364	0.318	0.719	0.095	0.465
*F*		0.060	3.145 ^†^	3.271	7.682*	1.477	5.658 *

*Note: *† *p *< 0.10; * p < 0.05; ** p  < 0.01; *** p < 0.001. NVIQ entered in Step 1; WM Composite entered in Step 2. ^a^ Language measured using the Clinical Evaluation of Language Fundamentals, 4th Edition, Core Language (scaled score; [26]). ^b^ Symptom severity measured using: Social Responsiveness Scale [27] and Autism Diagnostic Observation Schedule (ADOS) Communication + Social Total Score [28].

### 2.3. Discussion

The current study explored spatial and verbal working memory (WM) abilities in children and adolescents with high-functioning autism and typical development. There have been conflicting reports on the nature of verbal WM skills in HFA [21]. In the current study, there was no difference in performance across a set of WM tasks addressing spatial, simple phonological verbal, and complex verbal WM skills; across tasks, the HFA group performed significantly worse than a comparison group matched on age, gender, nonverbal IQ, and, importantly, language abilities. The results suggest a domain-general WM limitation in HFA, consistent with prior reports of deficits in short-term phonological WM [14,15], spatial WM (e.g., [16,17]), and more complex verbal WM (e.g., [6,29]). Not only did participants with HFA display WM deficits relative to their TD peers, but over half of the HFA group was in the low average or clinically impaired range on at least one task. This suggests that WM difficulties are pervasive, clinically meaningful, and also likely to have a significant impact on real-world functioning.

Several factors may account for the finding of general WM limitations in the HFA group, unlike in prior reports. First, the current study carefully controlled for age, cognition, and language level—all factors that, separately, may partially explain prior discrepant findings. Second, our group with autism was well-defined, including only high-functioning children without Aspergers Disorder; many previous studies that have employed careful matching techniques may not have found similar results based on their inclusion criteria or comparison sample. For example, Williams, *et al*. [30] found that children with autism performed similarly to their peers on an immediate serial recall task. However, this was in comparison to peers that had learning disabilities, rather than those with typical development. Children’s cognitive abilities in both groups ranged from intellectually disabled to average, which may have masked more subtle group differences in executive functioning. 

The complex verbal WM tasks utilized in the current study required the ability to simultaneously maintain and manipulate multiple pieces of information, and hence relied more on executive processes than simple span tasks. For example, the competing language processing task (CLPT) relied heavily on linguistic processing and comprehension, thus drawing upon multiple systems including executive function *and* language processing. This is consistent with theories of complex functioning impairments in ASD [31], particularly those that suggest deficits become more apparent with increased executive function or language processing demands [4,21,32]. What can be more difficult to reconcile is the current study’s finding of impairments on simple WM tasks, in contrast to previous studies which failed to find group differences for verbal simple span tasks (e.g., [33,34,35]). Interestingly, a recent and carefully controlled study examining adults with HFA (defined as verbal IQ > 80) also found impairment on both simple digit and word span tasks [29]. Although individuals with autism recalled the information, they had greater difficulty than the control group in recalling this information in the correct order. Poirier and colleagues speculated that temporal processing may be impaired in autism. Simple WM is thought to reflect storage, or holding, while complex WM involves both storage and manipulation of information [19,36]. Temporal processing may be more essential in the storage aspects of WM, which would have implications for both simple and complex WM tasks. Given the global WM impairments noted in the current study, our results are consistent with Poirier, *et al*. [29], and support the suggestion that individuals with autism have difficulty in the “storage” component of WM common to both simple and complex span tasks.

There is also an alternative account for the current findings. Domain-general deficits in WM may be further exacerbated by domain-specific weaknesses. The two simple span tasks in the current study were either visuospatial or linguistic in nature. Visuospatial WM deficits, in comparison to verbal, are more consistently noted in ASD (e.g., [16,17]), suggesting that simple spatial span deficits found in the current study may be partially due to modality. In addition, phonological processing deficits are also noted in autism [14,15], suggesting that limitations in online linguistic processing may contribute to impaired performance on these tasks even though the WM demands were relatively simple.

The current study further explored associations between WM abilities, language, and social functioning in the HFA group. WM abilities accounted for a significant portion of variance in language skills, even when controlling for nonverbal IQ. Although consistent with the largely verbal nature of the WM tasks, this supports previous findings that suggest language and WM skills are closely intertwined [24]. The fact that language skills were closely tied to WM speaks to the importance of having carefully controlled samples.

WM also predicted social difficulty. Specifically, regression analyses indicated that WM ability, over and above nonverbal IQ, accounted for significant variance in symptom severity. This is consistent with previous studies linking symptom severity to WM skills in adults with HFA [37]. For successful social interactions, it is essential to maintain multiple pieces of information in mind at one time, and to update this information as necessary. One must keep track of information in a conversation [38], the body language of the conversational partner, and information about the interaction context. The relationship between WM ability and social skills in the current study suggests that both verbal and nonverbal aspects of WM may be relevant for this process.

The current findings expand on prior WM research in ASD. First, this study included both verbal and visuospatial WM measures within the same sample, to resolve discrepant findings in prior studies. Furthermore, our verbal WM tasks included a relatively simple phonological WM task as well as two more complex tasks, to test the possibility that verbal WM impairments in ASD may emerge as a function of increases in the executive or linguistic complexity of a task [4]. Our results suggest that the basic storage component of WM is implicated in autism, resulting in limitations in both simple and complex aspects of WM. In complex span tasks, this difficulty may become further exacerbated by greater executive or linguistic demands. Finally, due to the strong relationship between WM performance and language skills, one might conclude that group differences in WM may simply reflect weaknesses in language rather than WM performance. However, we would like to suggest an alternative conclusion: that language processing is, in many respects, a highly-demanding form of WM processing. The more complex the structure of language and discourse involved, the more apparent WM deficits may become. 

Perhaps most noteworthy, performance suggested a broad suite of WM deficits across tasks that require simple short-term storage (as is the case for simple phonological WM) and those that require updating and maintenance of information. Participant scores across the standardized measures also mapped onto caregiver report of executive functioning skills. Parents of all TD participants (*n *= 18) and a subset of HFA participants (*n *= 10) completed the Behavior Rating Inventory of Executive Function [39]. Caregivers in the HFA group endorsed significantly more symptoms on both the general composite measure of executive functioning, *F *(2, 25) = 62.74, *p *< 0.001, *η*^2^*_p_* = 0.71 (HFA group *M* = 70, *SD* = 8; TD group *M *= 45, *SD *= 8), as well as the specific WM subscale, *F *(2, 25) = 76.77, *p *< 0.001, *η*^2^*_p_* = 0.69 (HFA group *M* = 70, *SD* = 8; TD group *M* = 44, *SD* = 7). These scores suggest greater general WM challenges in the daily lives of children with HFA, and also indicate that these challenges are of clinical significance, as T-scores above 65 are considered to be clinically meaningful. Deficits in WM can lead to daily difficulties in multitasking, recalling what one needs to do, or completing tasks like homework. Furthermore, the WM deficits noted in the current study may also be reflective of more global difficulties in executive function, consistent with previous studies that have noted fluency [40] and attention-shifting [10,41,42] impairments in autism. 

The current study is limited in several dimensions. First, all of the standardized child assessments were examiner-administered. Some research has suggested that when tasks are computer-administered, particularly executive tasks, ASD-group deficits are less apparent [43,44]. It is clearly important to take into account the role of social interaction in WM performance. In addition, three of the four WM tasks were verbal in nature; while this afforded a thorough assessment of verbal WM, it prevented a more detailed examination of the interaction of language and nonverbal aspects of WM. Finally, groups were carefully matched on age, gender, nonverbal IQ, and language, and the ASD group included only children with high-functioning autism and Pervasive Developmental Disorder/Not Otherwise Specified (without Aspergers syndrome). Although these stringent criteria are strengths, they also limit the generalizability of results to more heterogeneous groups. It is imperative for future research to continue to explore WM profiles in both carefully controlled as well as more heterogeneous, community based samples. In addition, it will also be important to explore WM skills across the full autism spectrum.

## 3. Experimental Section

### 3.1. Participants

Participants included 18 individuals with high-functioning autism (HFA) or Pervasive Developmental Disorder/Not Otherwise Specified and 18 typically-developing (TD) controls, ages 8–17; individuals with Asperger Syndrome were excluded. Diagnoses were confirmed using the Autism Diagnostic Observation Schedule (ADOS; [28]), the Autism Diagnostic Interview-Revised (ADI-R; [45]), and expert clinical judgment based on DSM-IV-TR criteria. Participants had full scale IQ and standardized language scores of 80 and higher (as assessed by the Stanford-Binet, 5th Edition [46] and Clinical Evaluation of Language Fundamentals, 4th Edition (CELF-4; [26]), respectively). 

Groups were matched on chronological age, gender, and formal assessment measures of IQ and language. The HFA group was matched on nonverbal IQ to the TD group. In addition, there were no group differences in structural language abilities, as assessed by CELF-4 Core Language score, or in vocabulary level as assessed by the Peabody Picture Vocabulary Test, Third Edition [47] and Expressive Vocabulary Test, Second Edition [48]. Data are shown in Table 4. TD controls had no psychiatric, neurological, or behavioral impairments or family history of ASD, and their non-ASD status was confirmed using the Social Responsiveness Scale (SRS; [27]) and expert clinical judgment. Consent and assent were obtained prior to testing; procedures followed all human subjects guidelines.

**Table 4:** Participant demographics and standardized cognitive assessments.

		HFA *n* = 18 (16 boys)	TD Controls *n *= 18 (14 boys)	*χ^2^* or *F*	*p*	*η*^2^*_p_*
Age (years)		12 (3); 8–17	13 (2); 8–17	0.602	0.44	0.02
Full Scale IQ ^a^		105 (10); 94–127	104 (10); 88–127	0.11	0.75	0.01
	Verbal IQ	10 (3); 6–17	10 (2); 6–14	<0.001	1.00	<0.001
	Nonverbal IQ	12 (3); 7–17	11 (2); 8–16	0.65	0.43	0.02
CELF-4 ^b^		105 (13); 82–126	110 (7); 97–123	1.75	0.20	0.05
PPVT-3 ^c^		112 (13); 83–131	116 (12); 100–147	0.55	0.46	0.02
EVT-2 ^d^		107 (15); 81–136	109 (16); 84–139	0.10	0.75	0.01
SRS ^e^		81 (7); 71–90	40 (11); 0–57	104.29	<0.001	0.80

Notes: Data presented as Mean (SD); range. Results are standard scores, with a *M* = 100, *SD* = 15 or *M* = 10, *SD* = 3. ^a^ Stanford-Binet, 5th Ed., Abbreviated IQ [46]. ^b^ Clinical Evaluation of Language Fundamentals, 4th Ed. (CELF-4), Core Language [26]. ^c^ Peabody Picture Vocabulary Test, 3rd Ed. [47]. ^d^ Expressive Vocabulary Test, 2nd Ed. [48]. ^e^ Social Responsiveness Scale [27].

### 3.2. Measures

Working memory (WM) performance in three domains was assessed with four standardized measures. 1) *Simple spatial WM* was examined using the Finger Windows subtest from the Wide Range Assessment of Memory and Learning, in which the examiner placed a pencil into an increasingly-lengthy sequence of holes in a vertical plastic card, and asked the participant to reproduce the sequence. 2) *Complex Verbal WM* was examined using the Letter-Number Sequencing subtest from the Wechsler Intelligence Scale for Children, 4th Edition, in which the participant recalled a series of numbers and letters in alphabetical and numerical order; and with 3) a modified version of the Competing Language Processing Task (CLPT; [49]), in which the participant made true/false judgments for a series of statements, and then was asked to recall the final word of each statement. Finally, 4) *Simple Phonological WM* was examined using a non-word repetition task [50], in which the participant repeated nonsense words of increasing complexity. These tasks were completed over two sessions as part of a larger battery. In addition to comparison of WM scores across groups via repeated-measures ANOVA, we examined factors influencing WM performance (cognition, language, and ASD symptom severity) using linear regression. 

## 4. Conclusions

Clear WM impairments were noted in the HFA group across visuospatial, simple phonological, and complex verbal tasks. WM ability predicted both language skills and ASD symptom severity. These findings extend previous research suggesting WM impairments in ASD. While impairments in spatial WM have generally been consistently documented, the nature of verbal WM in ASD has been more controversial. Perhaps most striking in the current study was the generally poor performance of the HFA group across domains of WM, even when carefully controlling for age, cognition, and language. The strong relationship between WM and language and autism symptomatology offers insights for clinical intervention; it supports previous studies that suggest benefits to directly implementing WM demands into intervention through techniques such as rehearsal training or verbal WM drills [51,52].
